# Atomic Layer Deposition (ALD) of Alumina over Activated Carbon Electrodes Enabling a Stable 4 V Supercapacitor Operation

**DOI:** 10.1002/open.202000352

**Published:** 2021-02-15

**Authors:** Dayakar Gandla, Guanghui Song, Chongrui Wu, Yair Ein‐Eli, Daniel Q. Tan

**Affiliations:** ^1^ Guangdong Technion Israel Institute of Technology 241 Daxue Road, Jinping District Shantou Guangdong 515063 China; ^2^ Technion Israel Institute of Technology Haifa 3200003 Israel

**Keywords:** atomic layer deposition, supercapacitors, activated carbon, high voltage, self-discharge

## Abstract

Designing high voltage (>3 V) and stable electrochemical supercapacitors with low self‐discharge is desirable for the applications in modern electronic devices. This work demonstrates a 4 V symmetric supercapacitor with stabilized cycling performance through atomic layer deposition (ALD) of alumina (Al_2_O_3_) on the surface of activated carbon (AC). The 20‐cycle ALD Al_2_O_3_ coated AC delivers 84 % capacitance retention after 1000 charge/discharge cycles under 4 V, contrary to the bare AC cells having only 48 % retention. The extended cycling life is associated with the thickened Stern layer and suppressed oxygen functional group. The self‐discharge data also show that the Al_2_O_3_ coating enables AC cells to maintain 53 % of charge retention after 12 h, which is more than twice higher than that of bare AC cells under the same test protocol of 4 V charging. The curve fitting analysis reveals that ALD coating induced slow self‐discharge dominated by ion diffusion mechanism, thus enhancing the AC surface energy.

## Graphical abstract

**4 V symmetric supercapacitors** are stabilized through optimizing Atomic Layer Deposition of Al_2_O_3_ on activated carbon electrodes. The thickened Stern layer and suppressed oxygen functional group extend the cycling life at 4 V. The additional aluminum oxide layer enhances the energy barrier for ion desorption, thereby resulting in a decreased rate of self‐discharge. Too thick oxide coating blocks the nanopores and lower the cycling performance.

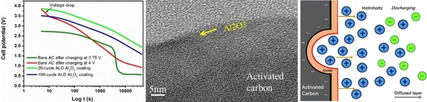

## 1 Introduction

In the last 20 years, the commercial electrochemical supercapacitor market is being dominated by activated carbonaceous materials (AC), because of their high surface area, high electric conductivity, temperature stability, excellent cycling stability, and low‐cost manufacturing.^[1]^ However, the AC electrodes suffer from low operating voltages (<3 V), high leakage current, resulting in a fast self‐discharge, inefficient charging/discharging cycles, and a significant amount of energy waste and heat generation when operated as a high‐power device.^[2]^ In order to enhance the energy storage capability of electric double layer capacitors (EDLCs), the sub‐nanometer carbon pores were utilized for higher capacitance, which led to the revisit of the helmholtz double‐layer model and ion diffusion.^[3]^ The fine‐tuning of the porous carbon structure and grafting electroactive molecules led to increased double‐layer capacitance (>200 F g^−1^ for aqueous electrolyte). However, attempts to increase the voltage window, energy density, and cycling stability failed.^[4]^ Recently, researchers utilized the dominant mesopore structures in bio‐mass derived ACs and demonstrated the feasibility of operating the device at potentials above 3 V, using conventional organic electrolytes.^[5]^ Yet, the complete picture of the ion transport and adsorption inside large porous networks in AC at higher potentials is not fully clear, while the need for further increase in operating potentials remains to be accomplished.^[6]^

An alternative way to increase the cell voltage is to stabilize the electrolyte and electrochemically active surface functional groups at the porous carbon surface. Using purer AC synthetic process to remove oxygen‐containing terminating groups from the porous carbon surface has led to introduction of commercial cells rated at 3 V. Passivating the surface of AC electrodes using a metal oxide much boosted the EDLC performance to 3.5 V in the lab.^[7]^ With an ultrathin Al_2_O_3_ using ALD, the commercial AC electrode exhibited an operating voltage of ∼3.5 V in EDLC cells with organic electrolytes.^[8]^ The ultrathin oxide coating effectively prevented the electrolyte‘s direct contact with the active sites on the carbons or the dissolution of the active materials, suppressing the decomposition of the electrolyte during the cyclic operation. When using mesoporous graphene sponge, the Al_2_O_3_ modification even enabled the graphene supercapacitor cells to reach up to 4.4 V.^[9]^ These studies encouraged a greater interest in further investigating the pathway towards higher voltage capability of the AC‐based EDLC device. In addition to suppressing the high voltage cycling deterioration issue, the self‐discharge is also yet to be addressed due to its critical impact in current EDLCs production.^[10]^ A high self‐discharge rate results in a significant and rapid voltage decay after charging, leading to a decreased energy and power.^[11]^ Therefore, it is of key importance to better understand the self‐discharge mechanism of supercapacitors (diffusion, Ohmic contact, overpotential/redox) operating at high voltages and to suppress the self‐discharge phenomenon.^[10]^

Resolving these technical issues will provide a significant understanding and will influence the introduction of ACs in high‐performance supercapacitor. In this communication, we report on surface modification of commercial AC electrodes by ALD of Al_2_O_3_ coating. We demonstrate that high cycle life and low self‐discharge operating at 4 V can be achieved without the use of high end and costly electrolytes.

## Experimental Section

Using commercial YP‐50F AC powder, the authors fabricated the AC electrodes on Al foils using the doctor blade technique through automatic thin‐film coater (MTI corporation). Later, it was vacuum dried for 12 h at 80 °C, and the thickness of the coated AC film was 100 μm. The ALD of Al_2_O_3_ was carried out using GEMSTAR XT^TM^ ALD system at 150 °C chamber temperature between 2 and 100 cycles using Trimethyl aluminum (TMA) and H_2_O (HPLC grade, Sigma‐Aldrich) as precursors at a constant argon gas flow rate of 10 sccm.^[8]^ During each deposition cycle, a pulsing time of 0.2 s and a purging time of 6 s was maintained, which gave rise to the typical growth rate of 1 Å per cycle for Al_2_O_3_.

## 2 Results and Discussions

### 2.1 Morphology Analysis

**Figure 1:**
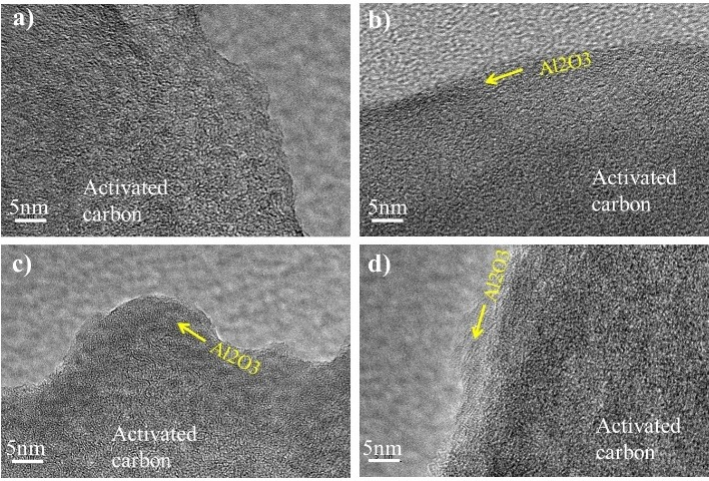
TEM images of AC electrodes coated with Al_2_O_3_ ALD of 2‐cycles **(a)**, 20‐cycles **(b)**, and 100‐cycles **(c)** and **(d)** showing thicker aluminium oxide with certain non‐uniformity.

The AC particle surface images of selected samples are inspected using a transmission electron microscopy (TEM), as shown in Figure 1. For a 2‐cycle ALD coating, the Al_2_O_3_ layer is barely observed on the surface of AC (Figure 1a); however, energy dispersion spectroscopy (EDS) already detects the presence of the aluminum element. With a 20‐cycle ALD coating, a thin layer of Al_2_O_3_ can be evident, as shown in Figure 1b, which appears to be on the order of a growth rate of ∼1 Å/cycle. With increasing ALD cycles, Al_2_O_3_ becomes thicker, as shown in Figure 1c and Figure 1d. However, the Al_2_O_3_ coating seems inhomogeneous and the EDS results confirm the variation of Al/O ratio. In all cases, the AC electrode surface chemistry is enriched with Al_2_O_3_ that may improve the surface energy state, suppressing the original oxygen functional group, and wettability for electrolyte.

### 2.2 Electrochemical Performance Analysis

The AC based supercapacitors suffers from the electrolyte decomposition during electrochemical cycling, especially at the high voltages and elevated temperatures due to undesirable parasitic side‐chain reactions between electrolyte and abundant surface‐active functional sites of electrode material.[^7b^, ^8^] Therefore, maintenance of cyclic life is the essential characteristic that needs to be ensured in new device development. It has been proven that the ALD surface modification is a unique technique to retain the cycle life of carbon‐based electrodes in organic electrolytes at higher working voltage, especially at 3 V. The ALD passivation of the carbon surface and its inhibition of the side‐chain reactions between the oxygen functional group of carbon and electrolyte may reduce the electrolyte breakdown at higher voltages.[^7b^, ^8^] However, there is no study to explain how the cycle life performance of carbon‐based supercapacitors gets influenced at voltages higher than 3.5 V. Therefore, we studied the cycle life performance of ALD coated AC using 1 M TEABF_4_/acetonitrile organic electrolyte at 3.5 V and 4 V (at a current density of 50 mA cm^−2^) by varying the thickness of ALD coating.

**Figure 2:**
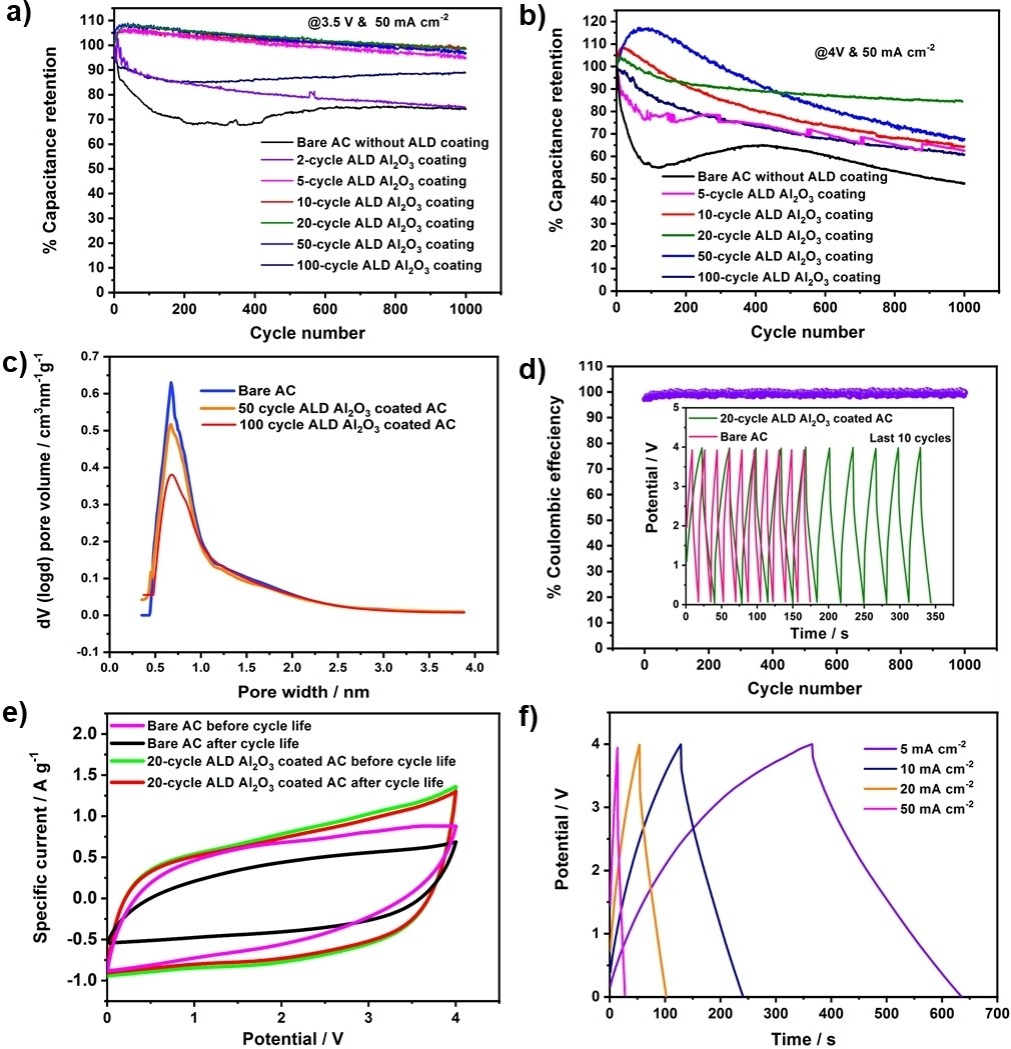
**(a)** and **(b)** Capacitance retention of the bare AC and ALD Al_2_O_3_ coated ACs at various ALD cycles measured at 3.5 V and 4 V, respectively, at a current density of 50 mA cm^−2^. **(c)** Pore size distribution by HK method. **(d)** Coulombic efficiency as a function of cycle number of 20‐cycle ALD coated AC. The inset shows the shapes of GCD curves during the last 10 cycles (990–1000^th^) of cycle life test at 4 V. **(e)** The shape of the CV curves of the bare AC and 20‐cycle ALD Al_2_O_3_ coated before and after cycle life. **(f)** GCD curves of the 20‐cycle ALD Al_2_O_3_ coated AC at various current densities from 5 to 50 mA cm^−2^.

At 2 cycles (∼0.2 nm), the improvement of retention shows to be limited and ends up at the same level as the bare AC electrodes (Figure 2a). However, 5 cycles of ALD coating quickly lifts the retention capability (pink line, Figure 2a), which shows the effect of thicker ALD coating. More ALD coating (10–50 cycles) stabilizes the retention performance at the optimum level (97 %), which prevents the electrolyte‘s reaction with the AC electrode during the electrochemical cycling more effectively. An extremely thick ALD coating at 100‐cycle lowers the capacitance; however it still maintains the cycling retention performance at 90 %, which implies the unnecessary coating and blocking of micropores. The optimum Al_2_O_3_ coating appears to be in the range of 10 to 50 cycles of ALD for enhanced capacitance retention, which did not block the AC‘s micropore channels. As is shown in Figure 2c, Horváth‐Kawazoe (**HK**) method for micropore size distribution analysis reveals that a similar pore width of 0.4 nm and peak position occur, although the peak intensity of bare AC is slightly decreased after 50 cycle ALD Al_2_O_3_ coating. This pore structure implies a minimal blockage of micropores until 50 cycles ALD coating, but not a significant blockage. Similar trend in cycle life performance upon increasing ALD cycle is observed at 4 V, where bare AC shows only 48 % capacitance retention till 1000 GCD cycles, whereas the AC cell with 20 cycles ALD shows the highest capacitance retention of 84 %. Further increasing the ALD cycle number beyond 20 cycles, the cyclic retention decreased, as shown in Figure 2b. This indicates that the cycle life of the AC cells trends lower in both directions of changing ALD cycles.

According to the Gouy‐Chapman‐Stern model,^[10e]^ the effective double‐layer thickness is reduced in nanopores (below 1–2 nm), rendering closer interactions of ions with carbon walls due to the pore size confinement. The nanopore‐dominated AC electrodes can′t handle very high voltages. The ALD coating modified the Stern layer as the sum of the additional coating and the original Stern layer defined by the ion radii and increased the voltage withstanding capability.^[8]^ For a non‐conformal and ultrathin coating thinner than 0.5 nm, the ions still arranged themselves in a similar configuration with a minor suppression of functional group. This effect resulted in less improvement in cycling performance where only 2‐cycles ALD coating is applied. With increasing the number of ALD cycles, a conformal layer not only suppresses the functional group, but also separates the electrolyte ions from the AC electrodes.^[7b]^ The spacer‐thickened Stern layer, along with the Debye diffuse layer, makes cells operate at higher voltages.^[8^] However, there exists no linear relationship between oxide thickness and operation voltage. When the oxide coating is too thick (10 nm), most of the nanopores are blocked, evidenced by the significant decrease in the pore volume (Figure 2c, red line). They are unable to contribute to the capacitance and thus result in the lower cycling performance again.

As shown in Figure 2d, during the cycle life test, 20‐cycle ALD modified AC shows a high coulombic efficiency of 99–100 %. When the shapes of the GCD curves are compared during the last 10 cycles, the 20‐cycle ALD Al_2_O_3_ coated AC shows the symmetrical charge‐discharge curves with a high charge‐discharge duration. In contrast, the bare AC shows a significant decrease in the charge‐discharge duration and slightly higher IR drop than the ALD modified AC, which implies a prominent loss of its capacitance. The coulombic efficiency of ALD coated AC is also shown to be high and stable over the cycling test. This kind of behavior is also confirmed by the CV curves (Figure 2e) as the bare AC showed a significant distortion and area reduction in the CV curves after cycle life test. However, the 20‐cycle ALD Al_2_O_3_ coated AC exhibits almost similar CV current and shape even after 1000 GCD cycles. The 4 V electrochemical performance of 20‐cycle ALD Al_2_O_3_ coated AC is measured at current densities ranging from 5 to 50 mA cm^−2^ (Figure 2f), which exhibits nearly symmetrical triangular shaped GCD curves implying its excellent electrochemical reversibility and good capacitive behavior.

### 2.3 Self‐Discharge Analysis

**Figure 3:**
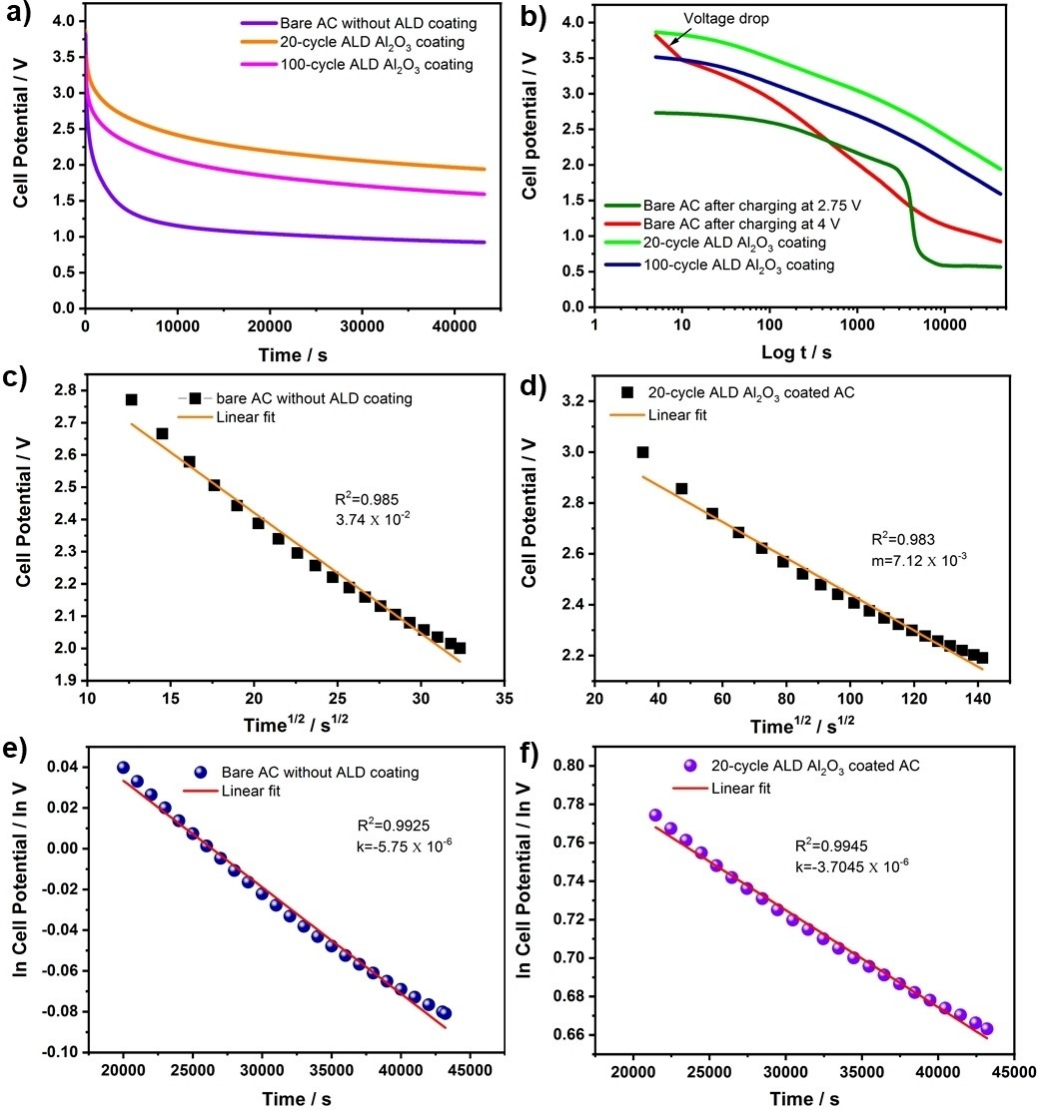
The self‐discharge mechanisms of the bare AC and ALD coated ACs **(a)** and **(b)** self‐discharge curves at linear and logarithmic scales with the dominant mechanisms of self‐discharge for each time region labelled. **(c)** and **(d)** fitted curves by the potential driving (Ohmic loss) model. **(e)** and **(f)** fitted curves by the diffusion‐controlled model.

Additionally, the charge storage capability of ALD coated AC cells at high voltages are investigated by measuring their open‐circuit potentials for 12 h after charging for 1 h at the constant voltage of 4 V. The bare AC maintains just 24 % of its initial potential, whereas the ACs coated with 20‐cycle and 100‐cycle Al_2_O_3_ show the enhanced charge retention of 53 % and 44 %, respectively, after discharging for 12 h (Figure 3a). A clear difference in the self‐discharge process of bare AC and ALD coated ACs is revealed when replotting the time in the log scale, as shown in Figure 3b. When charging the bare AC cell to 2.75 V, it does not exhibit any sharp drop in the cell potential at the initial stage (<10 s) of the self‐discharge process. However, a sharp drop in the cell potential occurs when charging the cell to 4 V. This overpotential decay is unwanted for a normal AC cell. However, the AC cells containing an ALD coating of 20 and 100 cycles Al_2_O_3_ did not show such behavior. Instead, they exhibited similar behavior as bare AC cell charged at 2.75 V, as shown in Figure 3b. This is attributed to the introduction of an additional Al_2_O_3_ layer on the AC surface, which thickens the effective Stern layer to withstand higher operating voltages. Besides, the cell potential of ALD coated AC has not shown plateau at the end of the measured time (Figure 3b). In contrast, the bare AC cell charged at 4 V and 2.75 V began to plateau after about 10,000 seconds. While this is not the open cell voltage of 0 V, it would require significantly longer time for absolutely all charges to dissipate from the electrodes, and so the plateau can be viewed as essentially unchanged.

In EDLCs, the self‐discharge process is governed by three mechanisms. The overpotential, diffusion‐controlled, and Ohmic loss (potential driving) mechanisms and these are explained by the linear dependence of cell potential vs logarithmic time, cell potential vs square root time, and ln potential vs time, respectively. These mechanisms often co‐occur but dominate in different time regions.[^10c^, ^11^] Figures 3c–3f confirmed the self‐discharge due to diffusion controlled process and Ohmic leakage by the linear fitting of the obtained data, where the data of bare AC and 20‐cycle ALD Al_2_O_3_ coated AC are taken into consideration for the better comparison of the models.

To explain the diffusion‐controlled mechanism, the self‐discharge curves display a linear relation between V and t^1/2^ according to Eq. 2:^[12]^

(1) $$V=V{}_{0}\text{-}\mathrm{mt}{}^{1/2}$$

**Figure 4:**
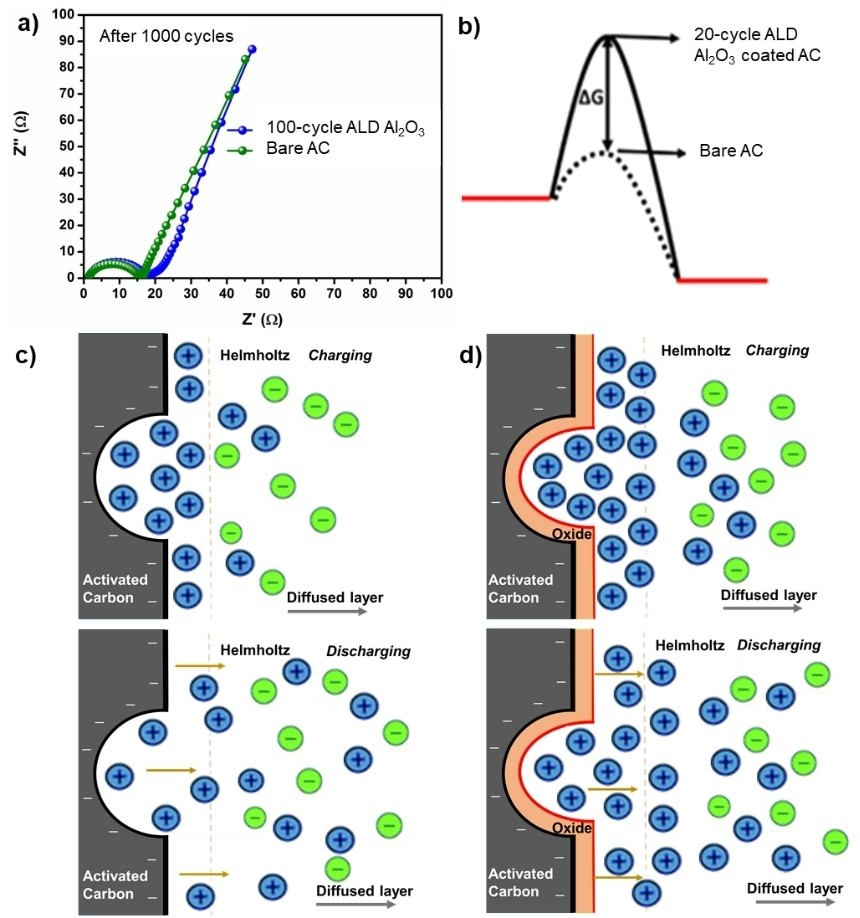
**(a)** EIS comparison of AC electrodes with and without Al_2_O_3_ coating after cycling tests, **(b)** energy profile and ion distribution view for the desorption of a TEA^+^ ion from organic electrolyte with (solid line) and without (dotted line) ALD Al_2_O_3_; Schematic models of ion distribution during charging and self‐discharging with **(c)** and without **(d)** ALD Al_2_O_3_.

Where m is the diffusion parameter corresponding to the diffusion rate of the ions near the electrode surface. By fitting the data in Figure 3c and 3d, the obtained value of m for bare AC was 3.74×10^−2^ V s^−1^, which is much larger than that of 20‐cycle ALD Al_2_O_3_. This may be understood as the suppression and passivation of surface functionalities of AC by thickened ALD coating, which inhibits the undesirable faradaic redox reactions between ions and surface functionalities of AC. Electrochemical impedance spectroscopy (EIS) analysis was taken for AC electrodes with and without ALD Al_2_O_3_ coating to show how the coating affects the impedance. Figure 4a shows no significant increase in the impedance of AC cells due to 10 nm thick ALD Al_2_O_3_ coating (lower impedance observed for 2 nm thick Al_2_O_3_). Yet, the Al_2_O_3_ coating changed the AC surface chemistry and the diffusion path of ions with respect to the baseline AC.

During the charging process, the ions from electrolytes are adsorbed on the surface of the ACs until it gets fully charged. The ions have to overcome a free energy barrier displayed in Figure 4b in order to desorb from the electrodes. If the barrier is low (shown in dotted lines), then the ions can quickly desorb and causes high self‐discharge as exhibited by the bare AC (Figure 4c). However, if the energy barrier is high, it inhibits the ion desorption and thus the spontaneous self‐discharge.^[12]^ The presence of Al_2_O_3_ on the AC surface as shown in Figure 4d, both, prevents the overpotential drop from the direct contact of AC‐ions and strengthens the residence of ions on Al_2_O_3_ as revealed by our own XPS analysis.^[13]^

Self‐discharge based on ion desorption can be written as a chemical reaction of A→B, where A denotes the absorbed ion and B is the free ion (Figure 4b). This first‐order reaction has a rate constant (k ) and Gibbs free energy of activation (ΔG≠), which can be derived from the rate constant based on transition state theory using the Eyring equation:(2)
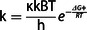



Where, 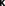 is the transmission coefficient (its value is equal to 1), kB the Boltzmann constant (1.38×10^−23^ J K^−1^), h the Planck constant (6.626×10^−34^ J K^−1^), R the universal gas constant (8.314 J mol^−1^ K^−1^) and T is the absolute temperature (293 K). The Ohmic leakage mechanism has the same linear relationship as for a first‐order reaction having a gradient equal to k. This rate constant (k) of bare AC and 20‐cyle ALD Al_2_O_3_ coated AC can be obtained from the slope as shown in Figure 3e and 3f. At 293 K, for bare AC, the value of k=5.75×10^−6^ s^−1^ yielded a ΔG≠=101,109 J mol^−1^, whereas the 20‐cyle ALD coated AC has the k=3.70×10^−6^ s^−1^ yielded a ΔG≠=102,180 J mol^−1^. Increase in the free energy of activation over 1 kJ mol^−1^ from ALD coating implies that the additional dielectric layer enhances the energy barrier for ion desorption, thereby results in a decrease in the rate of self‐discharge.

## 3 Conclusion

In conclusion, we have systematically investigated the ALD coating effect on the AC′s supercapacitor performance using cycle life, cyclic voltammetry, and galvanostatic charge‐discharge. We have achieved 4 V capability in AC‐based EDLCs through optimizing ALD thickness of Al_2_O_3_ on commercial AC surface. The investigation of ALD Al_2_O_3_ coating revealed the effective working thickness range of 1–5 nm (10–50 ALD cycles) for significantly stabilizing the cycling performance at 4 V. The enhanced voltage is attributed to the Al_2_O_3_ coating effect, however, is not proportional to the coating thickness. Too thick oxide coating (10 nm) blocked most of the nanopores and thus lowered the cycling performance of capacitance. The Al_2_O_3_ coating may act as the spacer separating the electrolyte ions from the AC, which not only suppressed the adverse effect of oxygen functional group on the original AC electrodes, but also enabled higher voltages through the thickened Stern layer. The self‐discharging experiment and data analysis indicated that ALD oxide coating could eliminate the overpotential issue for the AC electrode when operating at 4 V and slow down the ion diffusion rate. The ALD coating also increased the free energy of activation by 1 kJ mol^−1^ as compared to that of the bare AC cells, which may be attributed to the enhanced surface energy or electrolyte wettability against ion desorption.

## Conflict of interest

The authors declare no conflict of interest.

## Supporting information

As a service to our authors and readers, this journal provides supporting information supplied by the authors. Such materials are peer reviewed and may be re‐organized for online delivery, but are not copy‐edited or typeset. Technical support issues arising from supporting information (other than missing files) should be addressed to the authors.

Supplementary

## References

[1] R. Kötz , M. Carlen , Electrochim. Acta 2000, 45, 2483.

[2] C.-F. Liu , Y.-C. Liu , T.-Y. Yi , C.-C. Hu , Carbon 2019, 145, 529.

[3] M. Salanne , B. Rotenberg , K. Naoi , K. Kaneko , P. L. Taberna , C. P. Grey , B. Dunn , P. Simon , Nat. Energy 2016, 1, 16070.

[4] G. Pognon , T. Brousse , L. Demarconnay , D. Bélanger , J. Power Sources 2011, 196, 4117.

[5a] L. Suárez , T. A. Centeno , J. Power Sources 2020, 448, 227413;

[5b] X. Song , X. Ma , Y. Li , L. Ding , R. Jiang , Appl. Surf. Sci. 2019, 487, 189;

[5c] D. Gandla , H. Chen , D. Q. Tan , Mater. Res. Express 2020, 7, 085606.

[6] P. Simon , Y. Gogotsi , Nat. Mater. 2020, DOI: 10.1038/s41563-020-0747-z. 10.1038/s41563-020-0747-z 32747700

[7a] D. Q. Tan, R. A. Zhao *USA Patent 0002956 A1*, **2014**;

[7b] K. Hong , M. Cho , S. O. Kim , ACS Appl. Mater. Interfaces 2015, 7, 1899. 25548826 10.1021/am507673j

[8] D. Q. Tan , G. Song , D. Gandla , F. Zhang , Front. Energy Res. 2020, 8,596062.

[9] K. Nomura , H. Nishihara , N. Kobayashi , T. Asada , T. Kyotani , Energy Environ. Sci. 2019, 12, 1542.

[10a] B. W. Ricketts , C. Ton-That , J. Power Sources 2000, 89, 64;

[10b] M. Xia , J. Nie , Z. Zhang , X. Lu , Z. L. Wang , Nano Energy 2018, 47, 43;

[10c] M. Kaus , J. Kowal , D. U. Sauer , Electrochim. Acta 2010, 55, 7516;

[10d] H. A. Andreas , J. Electrochem. Soc. 2015, 16 2, A5047;

[10e] J. Lyklema , Fundamentals of Interface and Colloid Science, Academic, New York 1995.

[11] Y.-Z. Wang , X.-Y. Shan , D.-W. Wang , H.-M. Cheng , F. Li , J. Energy Chem. 2019, 38, 214.

[12] B. E. Conway , W. G. Pell , T. C. Liu , J. Power Sources 1997, 65, 53.

[13] G. Song , D. Q. Tan , Macromol. Mater. Eng. 2020, 305, 2000127.

