# Supplementary material for: Atomic Layer Deposition (ALD) of Alumina over Activated Carbon Electrodes Enabling a Stable 4 V Supercapacitor Operation
Source: ChemistryOpen. 2021 Feb 15;10(4):402–7. doi: 10.1002/open.202000352 (PMC8015731; doi:10.1002/open.202000352)
Supplement: Supplementary file 1 — Supplementary [file OPEN-10-402-s001.pdf]

ChemistryOpen

Supporting Information

Atomic Layer Deposition (ALD) of Alumina over Activated Carbon Electrodes Enabling a Stable 4 V Supercapacitor Operation

Dayakar Gandla, Guanghui Song, Chongrui Wu, Yair Ein-Eli,* and Daniel Q. Tan*

Supporting Information

1. Materials characterization

Transmission electron microscopy (TEM) images were captured on a JEM2100 instrument at an acceleration voltage of 200 kV. The pore size distribution analysis of the samples was carried out by Horváth-Kawazoe (HK) method through N_2 adsorption-desorption isotherms by Quantachrome Autosorb-iQ2-MP (USA) Nova-1000 system at -196 °C. The degassing of the samples was performed at 250 °C for 3 h.

2. Electrochemical characterization

The ACs' supercapacitor performance was evaluated through CR2032 coin cells using the gamry electrochemical workstation (Interface 1010E, USA). The electrodes were vacuum dried at 50 °C for 12 h, then, they were assembled in a glovebox by maintaining water and O_2 levels <0.01 ppm. 1M TEABF4/acetonitrile was used as electrolyte. A 35-μm thick cellulose NKK TF4035 (Nippon Kodoshi, Japan), having 75% porosity was used as a separator. Aluminum foil with 20 μm in thickness was used as a current collector. The slurry of AC electrodes was prepared by mixing of YP-50F AC powder (∼85%), super P carbon black (∼10%), and polyvinylidene difluoride (∼5%) in N-methyl-2-pyrrolidinone solution, and then a vortex mixer was used for 15-30 min to achieve a homogeneous mixing. The carbon slurry was then spread onto a sheet of current collector (aluminum foil) with a doctor blade technique to produce a homogenous film. Afterward, this coated film was vacuum dried at 80 °C for 12 h, and the final thickness of the coated AC was ∼100 μm. Finally, it was pressed to a dense sheet at 10 MPa and ALD was performed on these electrodes. The loaded active mass on each working electrode was found to be ∼3 mg cm^{-2}. Galvanostatic charge-discharge (GCD) measurements were carried out between 5 and 50 mA cm^{-2}. The cycle life test was performed at a current density of 50 mA cm^{-2}. EIS data were recorded with a 5 mV amplitude potential within the frequency range of 10 kHz-0.01 Hz.
